# Indigenous knowledge and modern science: a comprehensive review of phytochemicals in *Panax ginseng* flower for nutritional and health applications

**DOI:** 10.3389/fnut.2026.1857685

**Published:** 2026-06-19

**Authors:** Siqi Guo, Haiqing Luo, Shuxian Yang, Jiali Guan, Li Li, Meng Wang, Hilola Ahunova, Komiljon Tojibaev, Mengmeng Sun, Min He

**Affiliations:** 1 Northeast Asia Research Institute of Traditional Chinese Medicine, Changchun University of Chinese Medicine, Jingyue Economic Development District, Changchun, China; 2 The Jilin Province School-Enterprise Cooperation Technology Innovation Laboratory of Herbal Efficacy Evaluation Based on Zebrafish Model Organisms, Changchun University of Chinese Medicine, Jingyue Economic Development District, Changchun, China; 3 Affiliated Hospital of Changchun University of Chinese Medicine, Chaoyang District, Changchun, China; 4 Capital Medical University, Subsidiary Beijing Hospital of Traditional Chinese Medicine, Dongcheng District, Beijing, China; 5 Beijing Institute of Traditional Chinese Medicine, Shuiche Alley Xinjiekou, Xicheng District, Beijing, China; 6 Wish Technology, High-Tech North District, Changchun, China; 7 Institute of Botany, Academy of Sciences of Uzbekistan, Tashkent, Uzbekistan

**Keywords:** bioactive compounds, functional food, ginseng flower, ginsenosides, sustainable nutrition

## Abstract

*Panax ginseng* flowers, an annually renewable yet underutilized byproduct of ginseng cultivation, represent a promising raw material for functional foods that bridges traditional East Asian ethnobotanical practices with modern nutritional science. This review systematically summarizes the phytochemical constituents and pharmacological activities of ginseng flowers to evaluate their potential as health-oriented food ingredients. Published studies from recent decades were collected and analyzed. The major bioactive compounds identified include dammarane-type ginsenosides (protopanaxadiol and protopanaxatriol types), malonylated ginsenosides, polysaccharides, and volatile constituents. Ginseng flower exhibits a broad spectrum of bioactivities and health-promoting potential, including antioxidant, anti-inflammatory, immunomodulatory, anti-fatigue, hepatoprotective, and cardiovascular protective effects. In terms of food applications, ginseng flowers have been explored as functional ingredients in beverages and fermented foods, with preliminary studies confirming technical feasibility and initial consumer acceptance. However, several challenges limit their industrial application: thermal processing induces transformation or degradation of heat-sensitive ginsenosides; bitterness and astringency hinder consumer acceptance; bioactive variability depends on cultivar, growing conditions, and harvest stage; and high-quality clinical evidence remains insufficient. Ginseng flower holds significant potential as a sustainable functional food ingredient, and future research should focus on mild processing technologies, sensory optimization, and quality standardization to facilitate its evidence-based development.

## 1 Introduction

**Figure 1:**
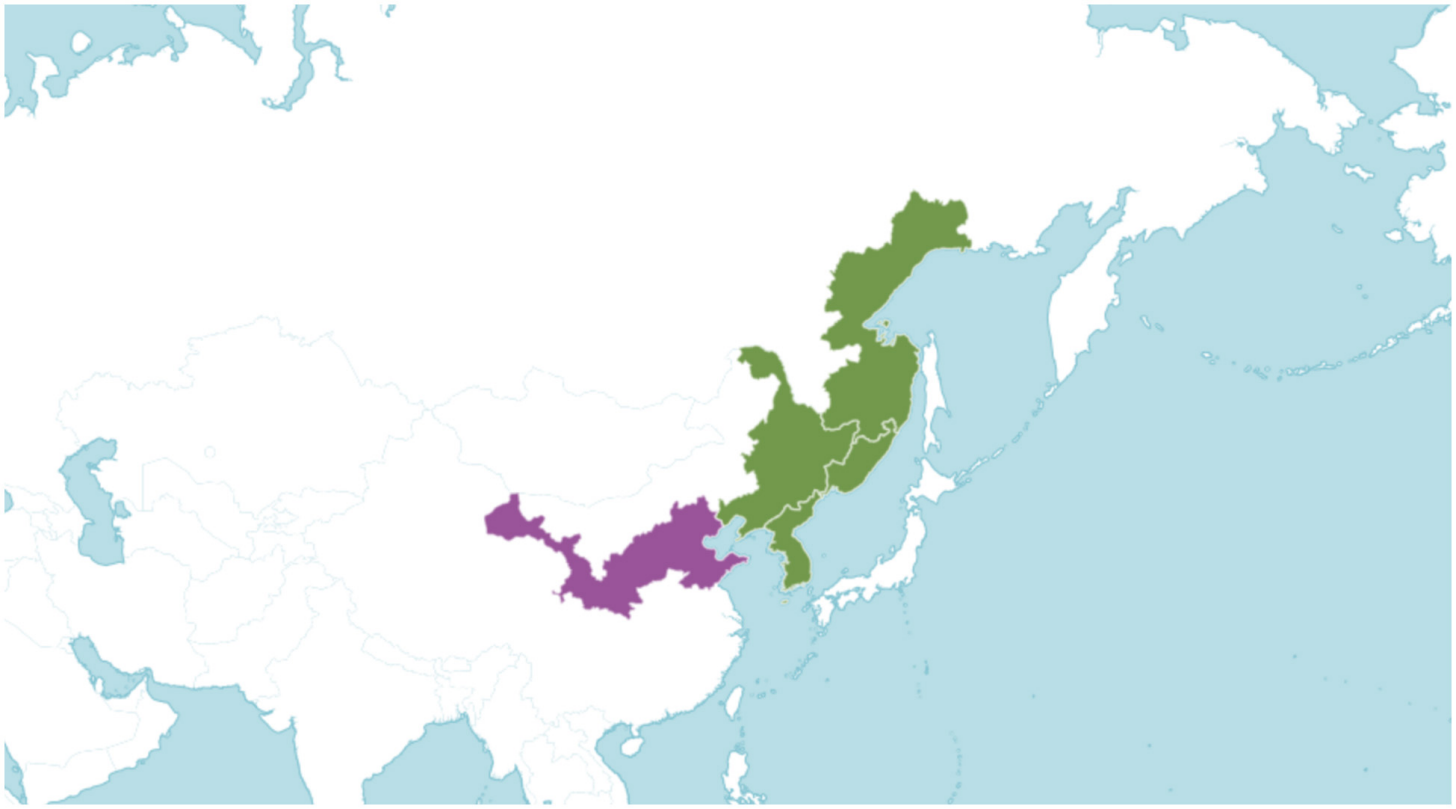
The geographical distribution of Panax ginseng C.A.Mey. Map data sourced from the World Checklist of Vascular Plants (WCVP), facilitated by the Royal Botanic Gardens, Kew. © Copyright 2023 World Checklist of Vascular Plants, licensed under CC BY 3.0. Taxon page: (https://powo.science.kew.org/taxon/urn:lsid:ipni.org:names:91472-1) (80).

*Panax ginseng* C.A. Meyer, a perennial herb of the genus Panax (Araliaceae) (1), is widely cultivated across East Asia, including China, Japan, and Korea, where it has a long history of both native distribution and introduction as shown in Figure 1. The plant typically grows to a height of 30–60 cm and possesses a fleshy, aromatic root that is often fusiform or cylindrical, sometimes branched. The stem is erect, unbranched, and bears a whorl of compound leaves at the apex. Each compound leaf is palmately divided into five (occasionally three to seven) leaflets, which are ovate to elliptic, finely serrate, and glabrous on both surfaces (2, 3). In traditional medical and dietary practices, ginseng has long been used to support homeostasis and to alleviate diverse ailments. Driven by its recognized health benefits (4), contemporary development has focused predominantly on the root, leading to a broad range of commercial products such as beverages, candies, teas, and honey slices (5–9). From the perspective of sustainable nutrition and functional-food innovation, however, this root-centered utilization leaves substantial value in the annually renewable aerial parts—stems, leaves, berries, and particularly flowers—which represent promising, underused sources of bioactive phytochemicals.

Ginseng flower is a single terminal umbel, emerging from the center of the leaf whorl, bearing 20–50 small, pale green to yellowish-green flowers, and is typically harvested each year in June-July (10). It has been traditionally valued for “strengthening” effects and is commonly associated with immune support, anti-aging, and detoxifying uses. Since research efforts began in the 1970s, evidence has accumulated that the flower, like the root (10, 11), it is rich in ginsenosides (12) and has a favorable safety profile for long-term consumption (13). Traditionally consumed as wine and tea infusions, it now appears in modern functional beverages often blended with fruit juices and xylitol, and has also been used in beer production (14–18). These evolving applications illustrate how indigenous/traditional consumption patterns can inform contemporary product design, while also underscoring the need for scientific clarification of composition-function relationships relevant to sustainable health-oriented foods.

Chemically, ginseng flower contains a diverse set of constituents, containing ginsenosides, volatile oils, fatty acids, polysaccharides, flavonoids, and peptides (19, 20). Beyond sharing common saponins with the root (21), it contains unique constituents such as ginseng bud nona-and undecapeptides, which help regulate intracellular cAMP-cGMP balance (22). Pharmacological studies confirm that ginseng flower exhibits bioactivities similar to the root, including anti-inflammatory, antioxidant, anti-fatigue, gastroprotective, and cardioprotective effects (23, 24). Together, these findings position ginseng flower as a multifunctional botanical resource with clear relevance to functional foods aimed at supporting resilience, metabolic balance, and overall health.

**Figure 2:**
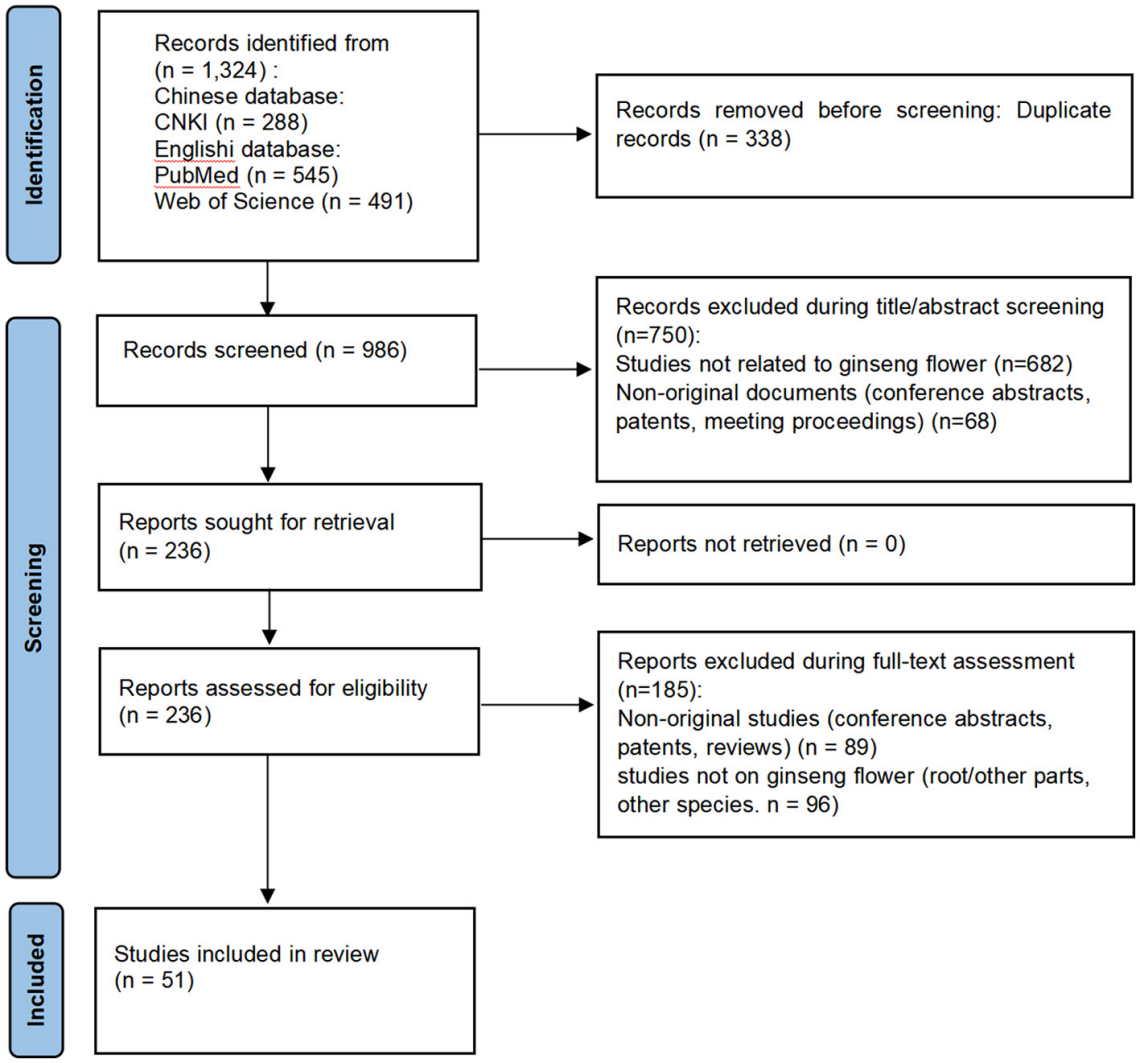
Flow diagram of the literature search. The literature was searched based on public databases, including Chinese database (CNKI) and English databases (PubMed and Web of Science), using the keywords “ginseng flower”, “*Panax ginseng* flower”, and “ginseng buds”. A total of 1,324 records were collected from the three databases: CNKI (*n* = 288), PubMed (*n* = 545), and Web of Science (*n* = 491). After removing 338 duplicates, 986 records underwent title/abstract screening, excluding 750 records (682 not related to ginseng flower, 68 non-original). The remaining 236 full-text articles were assessed, and 185 were excluded (89 non-original, 96 not on ginseng flower). Finally, 51 original studies were included with full author consensus. Consensus on inclusion was reached among the authors without any disagreement. This flowchart is adapted from the PRISMA template to illustrate the literature selection process for this narrative review; it does not indicate that a systematic review or meta-analysis was performed.

**Figure 3:**
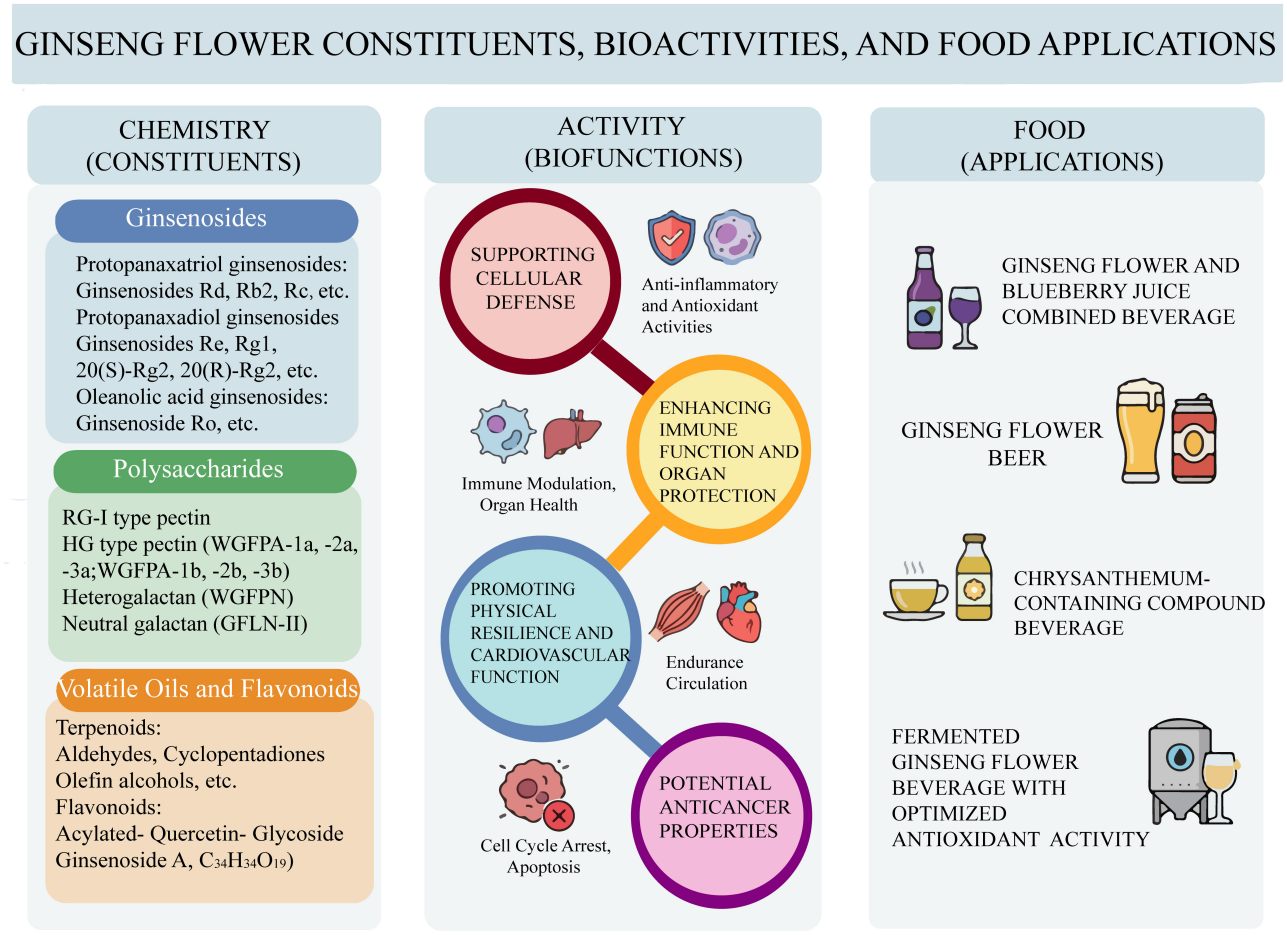
A schematic overview of the major chemical constituents, associated biological activities, and food applications of ginseng flower (Panax ginseng). **(Left)** Chemical components of ginseng flower; **(Center)** Biological activities of ginseng flower; **(Right)** Food applications of ginseng flower.

Despite this established knowledge base, translating ginseng flower from traditional use and laboratory findings into standardized functional ingredients remains challenging. Current evidence is often dispersed across studies emphasizing single compounds, single extraction conditions, or isolated bioactivities, limiting an integrated understanding that links its characteristic phytochemical profile to specific nutrition and health applications. In parallel, product development is constrained by practical hurdles such as the stabilization of key constituents (e.g., malonylginsenosides) during processing, sensory limitations (notably bitterness), and the need for stronger human evidence to substantiate health benefits and guide intake recommendations. Addressing these gaps is essential for aligning indigenous knowledge-driven uses with modern requirements for quality control, reproducibility, and clinical credibility in sustainable functional foods. Addressing these gaps is essential for aligning indigenous knowledge-driven uses with modern requirements for quality control, reproducibility, and clinical credibility in sustainable functional foods. To address these gaps, this review summarized the relevant information, which is structured into three sections: chemical constituents, functional activities, and food-related applications and products. This review enables readers to comprehensively synthesize current findings on the chemical constituents, extraction methods, and demonstrated functional potentials of ginseng flower. Additionally, this review also enables readers to evaluate the translational considerations and potential pathways of ginseng flower for its application in functional products. Following a protocol of literature collection, this narrative review collected 51 articles for our further synthesis (Figure 2). Figure 3 provides a schematic summary of the major chemical constituents of ginseng flower, their associated biological activities and potential food applications, offering a framework for the detailed discussion that follows. Through this approach, the present work aims to outline a forward-looking research and development framework. It seeks to identify key areas for further investigation, such as elucidating structure-activity relationships, refining stabilization and processing technologies, and advancing clinical validation. The ultimate goal is to support the evolution of ginseng flower into a well-characterized, evidence-informed botanical resource suitable for sustainable, health-promoting applications.

## 2 Chemical components of ginseng flower: a foundation for bioactivity and application

The chemical composition of ginseng flower is notably diverse and serves as the material basis for its purported health benefits. It encompasses several major classes of bioactive compounds, including saponins (ginsenosides), polysaccharides, volatile oils, and flavonoids, as systematically summarized in Table 1. It provides a systematic summary of the specific constituents identified within each class, along with the key extraction, separation, and analytical techniques used for their characterization. Among these, ginsenosides have been studied most extensively and are widely regarded as the principal bioactive constituents, largely dictating the flower's pharmacological profile. The following sections detail the characteristic components within each class, highlighting their structural uniqueness and relevance to the functional potential of ginseng flower.

**Table 1:** Major chemical constituents identified in ginseng flowers and their analytical characterization.

Compounds	Structure type	Extraction and separation methods	Experimental methods/techniques	References
51 ginsenosides (e.g., Re, Rg1, Rf, etc.)	Dammarane type, oleanolic acid type, etc.	70% methanol ultrasonic extraction^a^	UFLC-Triple TOF-MS/MS, UFLC-QTRAP-MS/MS^b^ (multiple reaction monitoring)	(26)
32 ginsenosides (e.g., Rb1, Rf, etc.)	Dammarane-type triterpenoid saponins, oleanane-type, malonylated PPD-type saponins, orcrin-type (OT-type)	70% methanol ultrasonic extraction	UPLC-MS/MS^b^	(27)
21 dammarane saponins (e.g., Re, Rg1, etc.)	Dammarane saponins, malonylated saponins	70% methanol ultrasonic extraction	UPLC-MS/MS^b^	(28)
14 ginsenosides (e.g., Rb1, Re, Ro, etc.) and 5 malonyl ginsenosides (e.g., mRb1, mRc, mRd, etc.)	Dammarane triterpenoid saponins (PPD type, PPT type), oleanolic triterpenoid saponins, and malonyl-damarane triterpenoid saponins	Boiling water, ethanol reflux and methanol extraction method^a^	RRLC-Q-TOF-MS^b^	(29)
8 ginsenosides (Re, Rd, Rf, etc.), 3 saponin aglycones	Dammarane triterpenoid saponin	Ethanol reflux extraction, macroporous resin chromatography, MCI silica gel column chromatography, silica gel column chromatography, preparative HPLC purification^a^	ESI-MS, ^1^H-NMR, ^13^C-NMR^b^ structural identification	(30)
8 ginsenosides (Rg1, Re, Rb1, etc.)	Dammarane triterpenoid saponin	IL-UAE combined with the two-water phase system (ABS)^a^	HPLC, RSM^b^	(31)
19 malonyl ginsenosides (e.g., mRb1, mRb2)	Malonyl dammarane saponins	Ultrasonic extraction^a^	HPLC-MS/MS^b^	(32)
5 malonyl ginsenosides (mRe, mRb, mRb2, mRc, and mRd)	Malonyl dammarane saponins	80% methanol extraction, AB-8 resin, MPLC, and semi-preparative HPLC^a^	1D/2D NMR (^1^H-NMR, ^13^C-NMR, HSQC, HMBC, and ROESY), ESI-MS, HRESIMS, IR, acid hydrolysis + GC for sugar composition, TLC^b^	(33)
20(S)-methoxyginsenoside Rg3, 20(R)-methoxyginsenoside Rg3	Dammarane triterpenoid saponin	Ultrasonic extraction, Diaion HP-20 resin column, SPE, and semi-prepared HPLC^a^	NMR, HR-ESI-MS, HPLC-DAD^b^	(34)
5 low-polarity ginsenosides (Rg6, F4, Rk3, etc.)	Dammarane triterpenoid saponin	Heat treatment, citric acid treatment	HPLC, NMR^b^	(35)
Ginsenoside F5, Ginsenoside F3	Dammarane-type triterpenoid saponins	Semi-preparative reversed-phase HPLC (Daisogel C-18 column, gradient acetonitrile-water elution)^a^	NMR (^1^H, ^13^C), HR-ESI-MS, HPLC^b^(peak area normalization)	(36)
Ginsenoside I and II	Dammarane saponins (containing 24-hydroperoxyl groups)	70% ethanol extraction, D101 macroporous resin, silica gel column chromatography, HPLC preparation^a^	NMR (^1^H, ^13^C, HMBC, NOESY), HRFABMS, ESI-MS^b^	(37)
Ginsenoside III	Dammarane saponins (containing ketone groups)	70% ethanol extraction, macroporous resin, silica gel column chromatography, reversed-phase HPLC^a^	NMR (^1^H, ^13^C, DEPT, COSY), ESI-MS, Acid hydrolysis analysis^b^	(38)
Pectin (WGFPA-1a, −2a, −3a) Pectin (WGFPA-1b, −2b, −3b)	RG-I Pectin, HG Pectin	Ion exchange chromatography, size exclusion chromatography^a^	Enzymatic hydrolysis, ^13^C NMR, bio-layer interferometry^b^	(42)
WGFPN (A neutral polysaccharide)	Heterogalactan	Water extraction and alcohol precipitation, ion exchange column chromatography^a^	Phenol-sulfuric acid method, meta-hydroxybiphenyl method, Bradford method, HPLC method, FT-IR spectroscopy, NMR spectroscopy, and HPAEC^b^ analysis	(43)
GFLN-II	Neutral galactan	Water extraction and alcohol precipitation, DEAE cellulose column chromatography, ethanol fractionation, Sepharose CL-6B column chromatography, TSK-Gel G3000PW HPLC^a^	Monosaccharide composition analysis (HPLC), molecular weight determination (GPC), infrared spectroscopy, high-potassium permanganate oxidation, Smith degradation, methylation analysis, ^13^C NMR^b^	(44)
Hexanal, 2-methyl-1,3-cyclopentanedione, nonanal, (E)-2-octen-1-ol, 4-hydroxy-3-methyl-2-propenyl-2-cyclopentenone, isopulegol	Aldehydes, cyclopentanediols, olefin alcohols, cyclopentenones, monoterpenols	Extraction of volatile oils by steam distillation, direct detection by SDAPCI-MS^a^ (without chromatographic separation)	SDAPCI-MS (cation mode, [M+H]^+^), MS/MS analysis, GC-MS^b^ data comparison (NIST 05 database)	(48)
Cubene, Elemene, Spathulenol, Bergamotene, Phytone	Pentatene, pentatene alcohols, monoterpene, ketones	SPME-GC-MS^a^ (PDMS-DVB 75°C)	SPME-GC-MS^b^, Electron ionization source	(49)
β-farnesene, β-elemene, germacrene D, caryophyllene, phytol	Pentatene, diterpene alcohols	SD, SFE^a^	GC-MS^b^, mass spectrometry EI source, NIST 05 database comparison	(50)
Floralpanasenoside A	Acylated quercetin glycoside	Silica gel column chromatography, MCI gel column chromatography, semi-preparative HPLC^a^	NMR, HR-ESI-MS^b^, Molecular docking	(51)

^a^Compounds were identified using various extraction and isolation techniques, including ultrasonic-assisted extraction, steam distillation, and various chromatographic methods (e.g., column chromatography, HPLC, MPLC, and size-exclusion chromatography).

^b^Structural characterization was performed using mass spectrometry (ESI-MS, HR-ESI-MS, QTOF-MS, GC-MS) and nuclear magnetic resonance spectroscopy (1D/2D NMR).

### 2.1 Ginsenosides: characteristic and structurally diverse saponins

**Figure 4:**
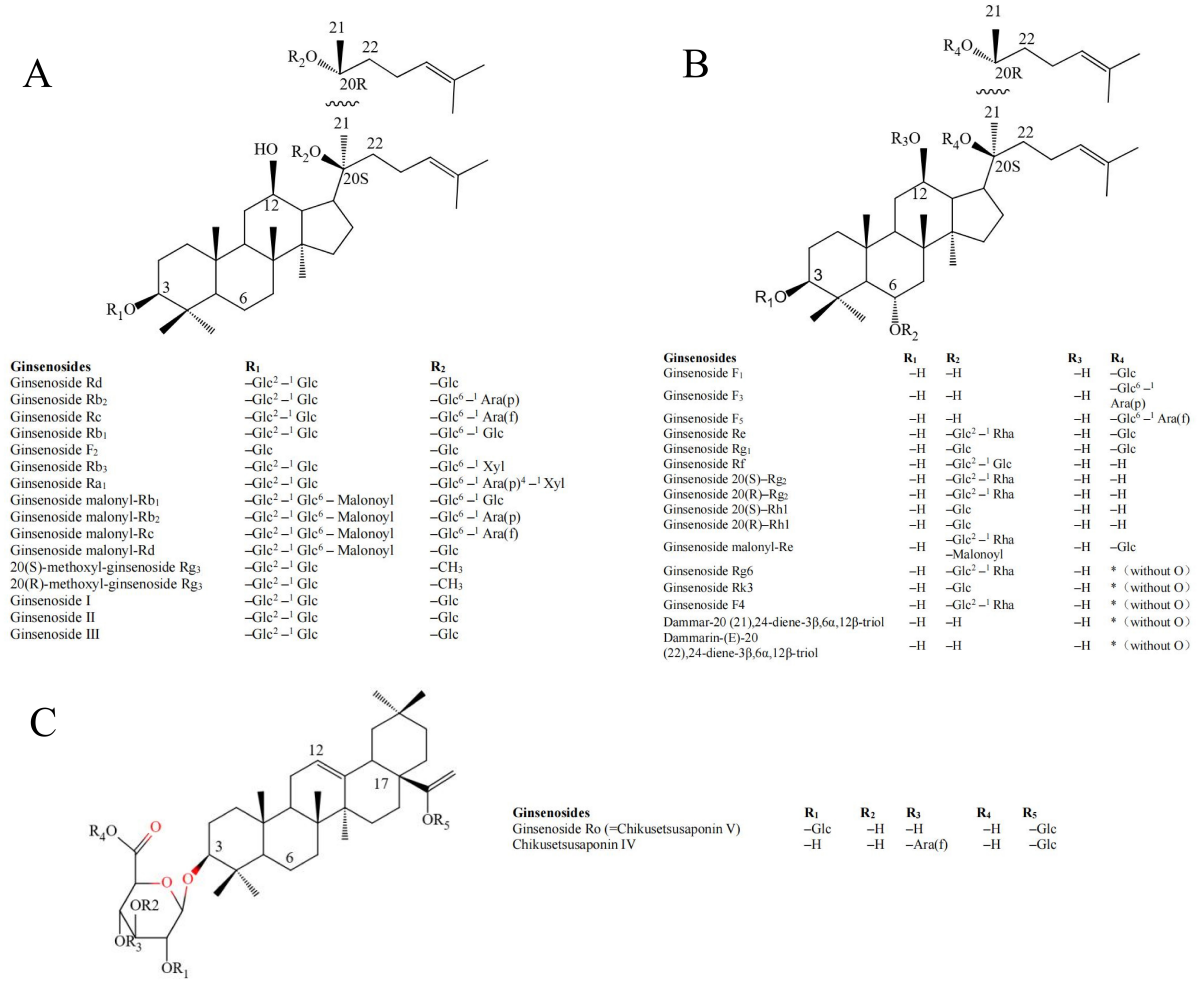
Characteristic aglycone structures found in ginseng flower ginsenosides. Shown are the representative scaffolds and substitution patterns for **(A)** protopanaxadiol (PPD) [*Ginsenoside I 24 (S or R) -COOH, 25 (26) C=C; *Ginsenoside II 24 (R or S) -COOH, 25 (26) C=C; *Ginsenoside III 24 -CHO, 25 (26) C=C], **(B)** protopanaxatriol (PPT) [*Ginsenoside Rg6, Ginsenoside Rk3, Dammar-20 (21), 24-diene-3β, 6α, 12β-triol: 20 (21) C=C; *Ginsenoside F4, Dammarin-(E)-20 (22), 24-diene-3β, 6α, 12β-triol: 20 (22) C=C], and **(C)** oleanolic acid (OA) types.

Ginsenosides represent the signature class of triterpenoid saponins in ginseng flowers. They are primarily classified based on their aglycone skeletons into three major types: protopanaxadiol (PPD), protopanaxatriol (PPT), and oleanolic acid (OA) (25). To date, over 60 distinct ginsenoside monomers have been isolated and identified from ginseng flower buds. Liquid chromatography-mass spectrometry (LC-MS) has been the cornerstone technique for their separation and structural elucidation (26). Figure 4 has mapped a comprehensive chemical profile, revealing a spectrum from common to rare structural types.

Common ginsenosides found in the flowers include Rb1, Rb2, Rd, Rc, Re, and Rf. Among these, Re, Rd, Rb2, and Rc are often reported as the most abundant, suggesting their utility as marker compounds for quality assessment (26–31). These common types span all three aglycone classes: PPD-type (e.g., Rd, Rb2, Rc, Rb1), PPT-type (e.g., Re, Rg1, Rf), and OA-type (e.g., Ro). Beyond these prevalent forms, the chemical landscape of ginseng flower is distinctively enriched with rare and structurally modified ginsenosides, which contribute to its unique bioactivity. A prominent feature is the abundance of malonyl-modified ginsenosides (e.g., malonyl-Rb1, malonyl-Rg3), which are prevalent secondary metabolites particularly associated with flower buds (32). Malonylation, involving the introduction of a malonyl moiety, can significantly alter the physicochemical properties and bioactivity of the base saponin. Numerous malonylated derivatives have been isolated, including novel compounds like a series of malonyl-Re, -Rb, -Rd, and -Rc isomers, with the first dimalonyl saponin in Panax plants being identified from this source (33).

In addition, structural diversity is further expanded by other modifications. Methoxylation, for instance, leads to distinct isomers such as the 20 (R)-and 20 (S)-methoxyginsenoside Rg3 pair, which exhibit differences in properties like solubility. Such low-polarity ginsenosides, including Rg6, F4, and Rk3, can be generated or enriched through specific processing methods like heat or acid treatment (34, 35). The structural complexity is augmented by various other rare saponins isolated from flower buds, such as ginsenosides F5, F3, the epimeric pair ginsenosides I and II, and the uniquely structured ginsenoside III (36–38).

In comparison with ginseng root, the flower shows a higher Rb1/Rg1 ratio (39) and abundant malonyl-ginsenosides (40), both of which can serve as marker compounds to distinguish the two organs.

### 2.2 Saponins polysaccharides: complex macromolecules with bioactive potential

Beyond ginsenosides, polysaccharides constitute another crucial group of bioactive macromolecules in ginseng flower, exhibiting notable bioactivities and structural complexity. While their structural elucidation presents distinct challenges (41), significant progress has been made. For instance, water-soluble pectic polysaccharides from ginseng flower buds were successfully resolved into six homogeneous fractions (WGFPA-1a to 3b) through combined ion-exchange and size-exclusion chromatography (42). Furthermore, a neutral polysaccharide designated WGFPN was characterized as an 11.0 kDa heterogalactan. Its unique architecture, featuring a (1 → 4)-β-D-galactan backbone with highly branched (1 → 6)-β-D-galactan side chains attached with arabinose residues, was elucidated *via* methylation analysis and 2D-NMR spectroscopy (43). In a separate study, fractionation of a crude polysaccharide (GFL) yielded a neutral fraction, GFLN-II, whose structural model was proposed with a backbone primarily of β-D-(1 → 4)-galactopyranose (44). These studies underscore the structural diversity of ginseng flower polysaccharides, which underpin their immunomodulatory and antioxidant activities discussed later.

Although less studied, the flower's polysaccharides differ structurally from root polysaccharides (e.g., more pectic arabinogalactans) (43, 45). No single marker exists, but linkage pattern differences could be used for differentiation.

### 2.3 Volatile oils and flavonoids: contributing to the phytochemical profile

The phytochemical profile of ginseng flower is complemented by volatile oils and flavonoids, which contribute to its overall sensory properties and bioactivity. Volatile oils, complex mixtures of aromatic compounds predominantly terpenoids, are present in ginseng flower (46, 47). Their analysis has employed various extraction techniques coupled with gas chromatography-mass spectrometry (GC-MS). Optimization of steam distillation (SD) facilitated direct analysis by a novel Surface Desorption Atmospheric Pressure Chemical Ionization Mass Spectrometry (SDAPCI-MS) method (48). Comparative studies show that supercritical CO_2_ extraction (SFE) offers a broader chemical profile than SD. Complementary approaches like Headspace Solid-Phase Microextraction GC-MS (HS-SPME-GC-MS) have further detailed the volatile composition, with one study collectively reporting 103 components (49). Concurrent analysis revealed a high proportion (58.62%) of unsaturated fatty acids, such as linoleic and linolenic acid, in the flower (50). Moreover, flavonoids, though less abundant, represent another significant class. Their discovery expands the known phytochemical diversity of ginseng flower. A novel acylated quercetin glycoside, Floralpanasenoside A, along with several known flavonoid glycosides, was isolated from the flower buds (51).

Unlike the root, ginseng flower contains detectable flavonoid glycosides (e.g., kaempferol-3-O-glucoside, Floralpanasenoside A) (51, 52) and a distinct fatty acid-rich volatile profile. Flavonoids are particularly useful marker compounds for distinguishing the flower from the root.

Collectively, the chemical differences between ginseng flower and root-especially the Rb1/Rg1 ratio, malonylated ginsenosides, and the presence of flavonoids-provide a basis for differentiating the two plant parts and for quality control of flower-derived products.

## 3 Bioactivities and health-promoting potential of ginseng flower

The diverse chemical profile of ginseng flower, encompassing its characteristic ginsenosides, polysaccharides, flavonoids, and volatile oils, underlies a broad spectrum of biological effects with significant implications for human health. Rather than being mere pharmacological observations, these bioactivities collectively define the flower's potential as a multi-functional botanical resource for health-promoting applications. They range from modulating fundamental processes like inflammation and oxidative stress to supporting specific organ functions and immune homeostasis. To translate these effects into tangible benefits, it is crucial to understand them within a health-oriented framework. The key experimental evidence from pivotal studies, including active components, models, and outcomes, is systematically summarized in Table 2. The following sections detail the evidence and mechanisms underpinning these major health-relevant activities.

**Table 2:** Summary of experimental studies on the biological activities of ginseng flower extracts and compounds.

Components	Dose	Model object	Modeling methods and drugs	Pharmacological activity	Duration of intervention	Index	References
Ginsenosides (Rg6, F4, ST1, SL2, SL3, Rh3, Rk2, and Rs4) in steamed ginseng leaves and flowers	5–50 μM	Bone marrow-derived dendritic cells (BMDCs)	LPS (10 ng/mL)	Anti-inflammatory	16 h	IL-12 p40 ↓	(55)
Ginsenosides (Rk3, Rs4, SF, and Rg6) in steamed ginseng flower	0.01–10 μM	HepG2, SK-Hep1 cell	TNF-α (10 ng/mL)	Anti-inflammatory	20 hours	iNOS, IL-8, CXCL1, ICAM1 ↓	(56)
Extraction of polysaccharides by M-GRPs (roots), M-GFPs (flowers), and M-GLPs (leaves) using multi-frequency ultrasound	0.5–2.5 mg/mL, 0.25–5 mg/mL	/	/	Antioxidant, α-glucosidase inhibitor	/	FRAP (Ferric Reoxidation Assay), DPPH free radical scavenging rate, superoxide anion scavenging rate, hydroxyl radical scavenging rate, α-glucosidase inhibition rate ↑	(78)
Water-soluble oligosaccharides from WGOS-R (root), WGOS-F (flower), and WGOS-L (leaf)	4.5 mL (0.1–0.6 mg/mL), 1.0mL (0.25–15 mg/ml), 1.0 ml (0.5–10 mg/ml)	/	/	Antioxidant	DPPH: 30 min; hydroxyl radical: 15 min; iron chelation: 10 min	DPPH radical scavenging rate, hydroxyl radical scavenging rate, and ferrous ion chelation rate ↑ SOD, CAT, GSH-Px, T-AOC ↑ MDA ↓	(60)
AHEP (acid extraction), DWEP (water extraction), ANEP (alkali extraction) of ginseng flower polysaccharides	1–10 mg/mL	/	/	Antioxidant, Cholesterol-lowering	DPPH/ABTS: 30 min; α-glucosidase: 30 min; bile acid binding: 1 h.	DPPH, ABTS clearance rate, bile acid binding capacity ↑	(61)
Ginseng flower water extract	1.6g/100g, 3.2g/100g, 6.4g/100g	BALB/c mice	UVB irradiation (total dose 2835 mJ/cm^2^)—skin photoaging	Antioxidant	Applied 30 min after irradiation for 6 consecutive days	Erythema and eschar scoring ↓ SOD ↑ MDA ↓ Nrf2 mRNA ↑	(62)
Ginseng flower water extract	1.0 g/kg	BALB/c mice	Cyclophosphamide (CTX, 50 mg/kg, i.p., days 26–29)	immunoprecipitation (significant)	30 days	Immune organ index (spleen, thymus) ↑ ConA induces splenocyte proliferation↑ DTH reaction ↑ leucocyte count ↑ Phagocytic function of macrophages ↑ NK cytoactive ↑ CD4+/CD8 + ratio ↑ Serum cytokines (IL-1β, IL-4, IL-6, IFN-γ, and TNF-α) ↑ Antioxidant protein ↑ (Nrf2, HO-1, NQO1, SOD1, SOD2, and CAT)	(64)
Ginsenoside Rd, Re	50, 100 mg/kg	Rat	Ethanol and indomethacin (20 mg/kg) induced gastric mucosal injury	Gastric protection	1 h after single administration	length of gastric mucosal injury ↓	(66)
Ethanol extract of *Panax ginseng* flower	300 mg/kg	SD rat	5% ethanol diet induces alcoholic fatty liver disease	Hepatoprotective	10 days	Fatty degeneration of liver tissue ↓ SREBP1c, FAS, SCD1, GPATGene expression of fat synthesis ↓ PPAR-α, CD36 Gene expression of lipolysis ↑	(67)
Ginseng flower extract (the formulation center may be related to the unique Rm7cd found in ginseng flower buds)	0.8g/kg, 0.6g/kg, 0.4g/kg	Mice	Fatigue induced by weighted swimming	Fatigue resistance	15 days	Weighted swimming time ↑ Blood lactate ↓ Muscle glycogen, liver glycogen ↑	(70)
Rb1	20 mg/kg	Mice	Intraperitoneal injection of reserpine (4 mg/kg) and 5-HTP (200 mg/kg) for drug-induced stress; Chronic Unpredictable Mild Stress (CUMS)	Anti-depression	30 min (acute) + 5 weeks (chronic)	BDNF, TrkB, AKT, ERK, and CREB protein expression ↑	(71)
Ginseng flower total saponins (Preparation Center)	*In vitro*: 40, 60, 80 mg/L; *in vivo*: 50, 100, 150 mg/kg	Wistar rat	*In vitro* working heart model (Langendorff perfusion method); coronary artery ligation to induce myocardial ischemia	Cardioprotective (enhances contractility, improves hemodynamics, reduces ischemic injury)	*In vitro*: within 20 min; *in vivo*: 21 days	AP ↑ LVSP ↑ ±dp/dtmax↑ cardiac output(CO) ↑ LVEDP ↓ HR ↓ CK ↓ LDH ↓ SOD ↑ MDA ↓	(72)
24(S/R)-ginsenoside M7cd, 24(S/R)-floralginsenoside Ka, 20(R/S)-ginsenoside Rh5	1–100 μM	HL-60, MGC80–3, Hep-G2 cell	/	Antineoplastic	48 h	IC50 (25.32–85.21 μM)	(73)
Floralginsenosides Ta-Tc, ginsenoside F1, F5	1–100 μM	HL-60 cell	/	Antineoplastic	24 h	Sub-G1 cell population ↑ apoptotic bodies formation ↑	(74)
6'-Malonylformyl ginsenoside F1 (1),3β-acetoxy ginsenoside F1 (2),7β-hydroxy ginsenoside Rd (8)	1–200 μM	HL-60, MGC80–3, Hep-G2 cell	/	Antineoplastic	48 h	IC50 (16.74–29.51 μM), (25.62–58.31 μM), (122.63–158.83 μM)	(75)
Coarse polysaccharide GF-2	10–100 ng/mL	Coriocyte	Progesterone ELISA Kit	Promotes progesterone secretion	4–24 h	Progesterone levels ↑	(79)

This summary provides information on the pharmacological effects of Panax ginseng flower (PGF) extracts and their purified constituents, using both *in vitro* (cell lines and cell-free assays) and *in vivo* (rodent strains) as experimental models. “↑” or “↓” illustrate the regulatory effects of PGF-derived components on different biological indicators.

### 3.1 Supporting cellular defense: anti-inflammatory and antioxidant activities

A key aspect of ginseng flower's health-promoting potential lies in its ability to support the body's cellular defense systems against inflammation and oxidative stress, which are fundamental to many chronic conditions.

*Modulation of inflammatory responses:* Beyond the well-documented anti-inflammatory properties of the ginseng root (53, 54), the flower, particularly after processing like steaming, serves as a notable source of active compounds. Ginsenosides Rg6 and F4, generated during steaming, significantly inhibited the production of interleukin-12 (IL-12), a key immunoregulatory cytokine, in LPS-stimulated cells (55, 56). Furthermore, dammarane-type ginsenosides (Rk3, Rs4, SF, and Rg6) from steamed flowers inhibited TNF-α-induced NF-κB activation in human liver cells and downregulated pro-inflammatory mediators like IL-8 and iNOS, with Rk3 and Rs4 showing the most pronounced effects (57). This modulation of critical inflammatory pathways highlights its potential role in managing chronic low-grade inflammation.

*Combating oxidative stress:* The antioxidant capacity of ginseng flower is primarily attributed to its polysaccharides and other constituents, which combat reactive oxygen species (ROS) (58, 59). This activity is not only a fundamental cellular protective mechanism but also a cornerstone for its broader health applications. Polysaccharides exhibit potent free radical scavenging abilities *in vitro*. *In vivo*, oligosaccharides from the flower (WGOS-F) enhanced systemic antioxidant defenses in aging mice by elevating SOD, CAT, and GSH-Px activities while reducing MDA levels (60). The extraction method critically influences this bioactivity; alkali-extracted polysaccharides (ANEP) showed superior antioxidant and α-glucosidase inhibitory activity compared to other methods (61). Topically, a ginseng flower ointment alleviated UVB-induced skin photoaging in mice, mechanistically linked to activating the endogenous Nrf2 antioxidant pathway (62).

### 3.2 Enhancing immune function and organ protection

Ginseng flower demonstrates a notable capacity to modulate immune function and support the resilience of specific organs, contributing to systemic well being.

*Immunomodulatory effects:* It enhances immune function through multiple mechanisms (63). In cyclophosphamide-induced immunosuppressed mice, ginseng flower extract up regulated key cytoprotective proteins (Nrf2, HO-1) in the spleen *via* the Nrf2 pathway, correlating with improved immune organ indices, a higher CD4^+^/CD8^+^ T cell ratio, and restored macrophage function (64). A recent study further supports that flower-derived compounds can mitigate immunosuppression and inflammation associated with metabolic stress (65).

*Gastroprotective and hepatoprotective actions:* Ginseng flower offers support for gastrointestinal and hepatic health. Bioactive components like ginsenoside Rd have shown significant protective effects against ethanol- and indomethacin-induced gastric mucosal damage in rats, with efficacy comparable to standard anti-ulcer drugs (66). For the liver, ethanol extract of ginseng flower ameliorated alcoholic fatty liver disease in rats by dual regulation of lipid metabolism: inhibiting lipogenic genes (SREBP1c, FAS) while activating those involved in lipid breakdown (PPAR-α, CD36) (67). Emerging evidence corroborates this; a study specifically highlighted the protective effects of ginseng flower extract against alcohol-induced liver injury, reinforcing its hepatoprotective potential (68).

### 3.3 Promoting physical resilience and cardiovascular function

The multifaceted adaptogenic properties of ginseng flower point to its broad application in supporting systemic resilience, encompassing physical energy metabolism, neural health, and cardiovascular function.

*Anti-fatigue and metabolic support:* Ginseng flower exhibits significant anti-fatigue properties by counteracting key metabolic drivers of exercise-induced fatigue. Physiologically, fatigue is associated with the depletion of muscle and liver glycogen reserves—critical energy sources—and the accumulation of lactic acid, a by-product of anaerobic glycolysis that disrupts metabolic homeostasis (69). Experimental studies demonstrate that ginseng flower extract directly addresses these hallmarks: in a mouse model, it prolonged swimming time, increased muscle and liver glycogen reserves, and reduced post-exercise blood lactate levels (70), indicating a role in enhancing physical adaptability and sustaining energy metabolism.

*Supporting neural health and stress adaptation:* Emerging evidence indicates that ginseng flower may also support neural health and stress adaptation, further extending its adaptogenic properties beyond physical fatigue. The flower contains abundant dammarane-type triterpene glycosides, notably ginsenoside Rb1. In a chronic stress mouse model, Rb1 markedly ameliorated depressive-like behaviors by activating the BDNF-TrkB-CREB signaling pathway, which is crucial for neuroplasticity. The mechanism involved enhancing the expression of brain-derived neurotrophic factor (BDNF) in key brain regions like the hippocampus and prefrontal cortex, along with activating downstream effectors including AKT, ERK1/2, and CREB (71). Notably, the water extract of ginseng flower demonstrated substantial antidepressant-like effects in behavioral tests, an activity linked not only to the aforementioned pathway but also to the regulation of monoamine neurotransmitter metabolism. This highlights its potential role in supporting mood balance and cognitive function under stress, aligning with its broader adaptogenic properties.

*Cardiovascular benefits:* Total saponins from ginseng flower demonstrated multi-target cardioprotective effects in an isolated heart model, improving myocardial contractility, attenuating oxidative stress (increased SOD, decreased MDA), and inhibiting platelet aggregation (72). These effects suggest potential in supporting cardiovascular function, possibly through modulating lipid metabolism and related signaling pathways.

### 3.4 Potential anticancer properties

Preliminary *in vitro* research reveals promising anticancer potential for specific ginsenosides isolated from ginseng flowers, primarily through inducing apoptosis. The stereochemical configuration is critical, with isomers having an S-configuration at C-20 or C-24 showing stronger anti-proliferative effects against human cancer cell lines (e.g., HL-60, Hep-G2) than their R-configured counterparts (73). Compounds like Floralginsenoside Ta and ginsenoside F5 induced apoptosis in HL-60 cells (74). Structure-activity relationship studies indicate that targeted modifications, such as acylation, can significantly enhance cytotoxic potency (75).

In summary, the health-promoting properties of ginseng flower extend beyond isolated effects. They collectively contribute to foundational support for resisting oxidative stress and inflammation, modulating immune and digestive health, promoting physical endurance and cardiovascular function, and include compounds with promising bioactive potential. This multi-faceted profile, rooted in its unique phytochemistry, underscores its value as a versatile botanical resource for nutrition and wellness. Translating this potential into practical applications requires targeted research, as outlined in the following discussion on challenges and future directions.

## 4 Functional food development of *Panax ginseng*

While the chemical diversity and pharmacological activities of ginseng flower form the scientific basis for its health benefits, the translational value of this botanical resource ultimately depends on its successful integration into functional food products. To date, several food applications have been explored to incorporate ginseng flower or its extracts into consumer-acceptable formats.

Product development efforts have included a health drink combining ginseng flower with blueberry juice (14), a ginseng flower beer (15), a compounded beverage with chrysanthemum (16), a fermented ginseng flower drink with optimized antioxidant activity (17) and a healthcare drink using ginseng flower and cider (18). Among these, only the fermented ginseng flower beverage was explicitly associated with antioxidant activity (17). The remaining examples primarily demonstrate that ginseng flower extracts can be successfully incorporated into various food matrices (alcoholic, non-alcoholic, fermented, non-fermented), suggesting their potential for further development into functional products.

Nevertheless, consumer acceptance studies on ginseng-containing food products have indicated that taste and sensory properties are critical determinants of success (5, 6). A recent study by Li et al. (7) analyzed the flavor volatiles and quality characteristics of ginseng products, providing a methodological framework for balancing bioactivity with palatability. Moreover, protein fortification has been shown to improve the physicochemical and sensory properties of yogurt supplemented with ginseng extract (9), suggesting that formulation strategies can overcome the bitterness commonly associated with ginsenosides.

In conclusion, while extensive research exists on the chemical constituents and bioactivity correlations of ginseng flower, only one product has been explicitly linked to a specific functional effect (antioxidant activity). Future efforts should prioritize translating the documented bioactivities into validated functional food applications.

## 5 Conclusions and future directions

This integrated review consolidates the current scientific evidence, positioning ginseng flower not merely as a by-product but as a distinctive and annually renewable botanical resource rich in bioactive potential. Its phytochemical profile, characterized by unique malonylated and less-polar ginsenosides, complex polysaccharides, flavonoids, and volatile oils, underpins a broad spectrum of health-relevant activities. These range from foundational support for cellular defense (anti-inflammatory, antioxidant) and immune modulation to organ protection (gastrointestinal, hepatic, and cardiovascular) and the promotion of physical and neural resilience. In the context of food applications, ginseng flower has been explored as a functional ingredient in beverages and fermented foods, with preliminary studies confirming technical feasibility and basic consumer acceptance. Critically, compared to the well-utilized root which requires years of cultivation, the annual harvestability of the flower presents a significant and underappreciated advantage for sustainable and scalable sourcing, aligning with the growing demand for eco-conscious functional ingredients. However, to transition this promise into tangible, evidence-based health products, several interrelated scientific and translational gaps must be strategically addressed. The development of ginseng-flower-based food products represents a particularly promising yet underexplored avenue for future research.

The primary challenge lies in bridging the gap between documented bioactivity and commercial viability. On the technical front, key hurdles include the inherent chemical instability of signature compounds (e.g., malonylginsenosides) during processing, and inherent sensory drawbacks like bitterness that hinder consumer acceptance in food formats—a challenge that may be addressed by novel formulation strategies (76). Furthermore, robust quality standards and safety specifications specific to flower-derived extracts are lacking. Scientifically, while the breadth of activity is promising, the depth of understanding remains limited. Current evidence heavily relies on conventional animal models and isolated compound studies, often overlooking the synergistic effects within the whole extract. The mechanistic underpinnings for systemic effects like anti-fatigue are not fully elucidated, and advanced analytical methods are needed to fully map the complex “compound-phenotype” relationships. From a functional perspective, among the various ginseng flower-based products developed to date, only one fermented beverage has been explicitly associated with a specific functional effect, namely antioxidant activity (17). The remaining product examples primarily demonstrate that ginseng flower extracts can be successfully incorporated into different food matrices, suggesting their potential for further development into functional products, although direct health claims have not been validated for those specific formulations.

To overcome these limitations and unlock the value of ginseng flower, a concerted, interdisciplinary research and development framework is proposed (Graphical Abstract). Future efforts must pivot toward application-oriented science. First, in cultivation and processing, research should focus on developing gentle, scalable stabilization technologies (e.g., combined physical field-assisted extraction) to preserve labile actives, alongside agronomic studies to optimize bioactive yield. Sensory optimization through food-grade strategies is crucial for product development. Second, biological research must evolve from phenomenological observation to mechanistic elucidation. Employing tiered model systems (77) from high-throughput platforms (e.g., zebrafish) to advanced mammalian models and *in vitro* organoids, coupled with multi-omics technologies (transcriptomics, metabolomics), will be key to deconvoluting mechanisms, synergies, and safety profiles. Finally, and most critically for translation, this foundational work must be conclusively bridged to human health. Rigorous, well-designed clinical trials are indispensable to substantiate physiological benefits and establish dosage. Concurrently, establishing comprehensive quality standards from farm to finished product is essential for regulatory approval and consumer trust. Future efforts must prioritize addressing these gaps through rigorous standardization, stability testing, and clinical evaluation. By explicitly linking chemical composition and bioactivity to tangible food applications, ginseng flower holds untapped potential as a value-added ingredient for the functional food industry.

In conclusion, ginseng flower emerges as a promising, sustainable, and multifaceted candidate for the next generation of health-promoting ingredients. Its successful evolution from a traditional botanical to a validated, market-ready functional resource hinges on a strategic pivot from pure phytochemistry to integrated application research. By uniting fields from agriculture and food science to molecular biology and clinical research, the potential of this annually renewable resource can be fully realized, offering novel solutions for human health while promoting the sustainable utilization of the entire ginseng plant.
